# Supplementary material for: Qualitative evaluation of barriers and facilitators to hepatocellular carcinoma care in North Carolina
Source: PLoS One. 2023 Jun 22;18(6):e0287338. doi: 10.1371/journal.pone.0287338 (PMC10287003; doi:10.1371/journal.pone.0287338)
Supplement: S1 Appendix — (PDF) [file pone.0287338.s001.pdf]

Draft Interview Guide

HCC-NC Qualitative Study: Interview Guide for UNC Patients

Greeting:
Hello, my name is _________ and I work with the University of North Carolina at Chapel Hill. I really appreciate you taking the time to participate in this interview.

Purpose:
I am part of a team working on a project about the experience of patients who have been diagnosed with liver cancer in North Carolina. You are being asked to participate in this study because you have been diagnosed with liver cancer. Please let me know if this is true and if you are willing to share your experiences with me in an interview. The information you will provide will assist us in understanding how we can best help patients with liver cancer. Are you willing to continue? (Note to interviewer: If yes, continue to next section. If not, thank participant and end interview).

Confidentiality and Introduction: To start, I'd like to stress that our team will keep everything said here today confidential. Your name will not be connected to what you say or your research file. Your doctor will not be told what you said. I hope that you will feel free to speak openly.

Please know that there is no right or wrong answer to these questions. My goal as an interviewer is to have you feel comfortable sharing your experiences, impressions, and beliefs to help us learn how to improve access to quality medical care for others diagnosed with liver cancer. Our discussion today will last about 45-60 minutes.

Before we begin, I would like to state that the conversation is being audio-taped to help us remember what is said during this discussion. You may ask me to turn off the recorder at any time or simply say you do not want to answer a question.

Do you have any questions before we begin?

Section I: Establishing a Context for liver cancer diagnosis (10 minutes)

1) To begin, please tell me a little about when you were diagnosed with liver cancer. (Allow time for them to answer open-ended question.)
    a. Who diagnosed you? Was this your regular medical care provider?
    b. How did things go when you received the news of your cancer diagnosis?
        i. During this conversation with your doctor, what worked or went well?
        ii. What could have gone better?
    c. Many patients with liver problems may never feel sick and may not know they have an underlying liver condition. Had anyone ever told you (before your cancer diagnosis) that you had underlying liver disease (such as hepatitis B or C, alcoholic cirrhosis, non-alcoholic fatty liver disease)?

2) Let's take a step back for a minute. Tell me about your medical care **before** being diagnosed with cancer.
    a. Prompts: How would you describe your relationship with your medical care provider?

Section II: Barriers to care (25 minutes) – Most important section

3) **KEY QUESTION: Have you seen a specialist for your liver cancer (such as a cancer doctor / oncologist, a GI doctor / hepatologist / liver specialist, a liver surgeon, or an interventional radiologist who treats liver cancer)?**
    i. [If yes] Where are you receiving your cancer care? (Which hospital / healthcare system?)
    ii. [If yes] Tell me about the process for getting an appointment with this specialist.
        1. What, if anything, went well?
        2. What, if anything, did not go well?
        3. Prompts: Getting the appointment scheduled, knowing what to expect about the appointment
    iii. [If no] Has anyone recommended that you see a specialist?

4) **KEY QUESTION: Once your diagnosis of liver cancer had been made, was there anything that has made it difficult to seek treatment or to see a specialist?**
    a. [If nothing offered, can prompt:]
        i. Transportation issues (financial or logistical)
            1. [If yes] About how long does it take you to drive from your home to your doctor's office?
        ii. Not feeling well enough to travel or attend an appointment
        iii. Other major medical problems
        iv. Other competing priorities/responsibilities (employment, childcare, care of a spouse or other family member)
        v. Co-pay / cost of visit
        vi. Delay in getting an appointment scheduled
        vii. Did not know which doctor or type of doctor I needed to see
    b. [If no difficulties expressed even after prompting:] I am glad to hear that you have not had any of these problems.

5) [This question is intended for those patients NOT already being seen at UNC, Duke or CMC in Charlotte] How far would you be willing to travel to see a liver cancer specialist?
    a. Tell me about what factors would impact your ability to travel to see a liver cancer specialist away from home.
        i. Are there any advantages or disadvantages, from your perspective, to being seen by a liver cancer specialist somewhere away from home?
        ii. Would you drive yourself or need someone to drive you?
        iii. Is the cost of travel a consideration?
        iv. Is the time away from home/work a consideration?

6) **KEY QUESTION: Tell me about the impact, if any, of the cost of your cancer treatment on you and your family.**
    a. Prompts:
        i. Is cost a source of worry or stress for you?
        ii. Is cost a consideration for you in making decisions about your treatment?
        iii. How has the cost of your cancer care affected your personal and family finances? (Prompt: Cutting back on spending on food, clothing, leisure activities)
        iv. Have you avoided any treatments due to cost? (Prompt: Not filling prescriptions for prescribed medications, taking less medication than prescribed, skipping office visits, deciding against treatments such as chemotherapy, surgery, or radiotherapy)
        v. Has anyone on your healthcare team discussed the cost of treatment with you?

Section III: Communication (10 minutes)

7) Communication with patients is very important.
    a. Since your cancer diagnosis, what instances, if any, have there been when you **did not feel** that your medical providers were communicating well **with you**?
        i. [If yes] Prompt: Tell me more about that. What could have been done differently?
    b. Is there a provider whom you identify as the "main provider" with whom you communicate for your liver cancer or whom you would contact if you needed help?
        i. [If yes] Who is that provider (type of provider)?
        ii. [if offered] Tell me about any experiences in contacting this provider.
            1. Prompts: Do you have a reliable way to reach a medical provider if you need help? Do you think you would be able to get a timely response?

8) Many patients with cancer want to hear from their doctors about what they can expect in the future. Tell me about anything that you might want to know about what to expect in the future.
    a. [If nothing offered, can prompt] Prognosis, survival, cancer-related symptoms
    a. I know these can be difficult topics to discuss. Did your doctor talk to you about any such things?
    b. Some people who are diagnosed with cancer want to be sure their "affairs are in order." Has this been something you have been thinking about?

<u>If time allows:</u>
    c. Some people choose to talk to family / friends about these issues.
        i. Have you talked to anyone (family, friends) about these things?
        ii. About your wishes for end-of-life care?

Section IV: Treatment (5 min)

9) I'd like to know more about your cancer treatment. Please tell me about any treatment that you have received for your cancer thus far.
   a. Possible types of treatment that patients may have received:
      i. Chemotherapy: "Chemo pills," sorafenib (Nexavar), IV chemotherapy
      ii. Surgery: Surgical resection to remove the part of the liver with cancer
      iii. Non-surgical localized therapy: Transarterial embolization, transarterial chemoembolization (aka TACE, beads, blockage), radiotherapy / radiation, radiofrequency ablation (RFA), radioembolization (TARE); non-surgical procedure using heat, a laser, radiation therapy, or by injecting a special alcohol directly into the cancer
   b. [If patient has received treatment] Tell me about your expectations for this treatment (good or bad).

   <u>If time allows:</u>

   c. [If patient has received treatment] Is there anything else about the treatment that you would like to know?

Section V: Daily functioning (5 min)

10) I'd like to talk to you about living with cancer. Many patients experience a change in their day-to-day activities as a result of their cancer or their cancer treatment. Have you experienced any such changes?
    a. Prompts: work duties, spending time with family/friends, caring for children/other family member, exercising, participating in activities in your community

11) Tell me about any people who have been sources of support to you and helped you since your diagnosis.
    a. Prompts:
       i. Spouse/partner, Child, Parent, Other family member, Friend
       ii. Church/religious organization, social worker, therapist, home-health aide
    b. What types of things are they helping you with?
       i. Prompts: emotional support, attending/transporting to appointments, ADLs (bathing, toileting, eating, dressing, walking), housework, meal preparation, etc.

Section VI: Closing (5 min)

12) Is there anything else that we did not cover that you would like to share with us?
13) Is there anything that your medical team could be doing to better care for you?

Thank you for your time today and for your willingness to participate in this study. You will be receiving a $40 gift card in the mail for your participation.
