# Supplementary material for: Qualitative evaluation of barriers and facilitators to hepatocellular carcinoma care in North Carolina
Source: PLoS One. 2023 Jun 22;18(6):e0287338. doi: 10.1371/journal.pone.0287338 (PMC10287003; doi:10.1371/journal.pone.0287338)
Supplement: S2 Appendix — (PDF) [file pone.0287338.s002.pdf]

HCC NC Phase I Qualitative Interviews (PI: Hanna Sanoff)

Research Question	Code Name	Description
**Establishing a Context**		
When were you diagnosed with LC?<br><br>Who diagnosed you?<br><br>How did things go when you received the news of your cancer diagnosis?<br><br>During this conversation with your doctor, what worked or went well?	**Pt_ diagnosis**	Use this code when patients talk about when they were diagnosed with liver cancer and by whom.<br><br>Use this code when patients talk about how they received the initial news that they had liver cancer and what about the conversation with their doctor they remember, good or bad.
Let's take a step back and tell me what your medical care was like before being diagnosed with cancer.<br><br>How would you describe your relationship with your medical care provider.	**Medical pre-LC diagnosis**	Use this code when patients talk about what their medical care or condition was like before diagnosis of liver cancer. May include discussions about Hep C, cirrhosis, conditions unrelated to the liver, etc. Use this code when patients talk about the relationship with their doctors prior to LC diagnosis.
	**LC care team**	Use this code when participants describe HCPs that are involved in their liver cancer care.
**Treatment**		
Once your diagnosis of liver cancer had been made, was there anything that has made it difficult to seek treatment or to see a specialist?	**Barriers to treatment**	Use this code when participants discuss post diagnosis challenges/barriers to receiving care. This could include transportation issue, feeling too sick, other medical issues, other competing priorities/responsibilities, appointment delays, etc.<br><br>Also apply this code when participants indicate that they have not experienced any barriers to treatment.
Tell me about the impact if any of the cost of your cancer treatment on you and your family?<br><br>Is cost a source of worry or stress for you?<br><br>Is cost a consideration for you in making decisions about your tx?<br><br>How has the cost of your cancer care affected your personal and family finances.<br><br>Have you avoided any treatments due to cost?<br><br>Has anyone on your healthcare team discussed the cost of treatment with you?	**Cost**	Use this code when participants describe anything related to the cost of liver cancer care.<br><br>This may include discussions about how cost may factor into receiving treatment.<br><br>Also use this code if participants discuss how the cost of cancer care has affected their personal finances or affected how they spend and save money.<br><br>Also use this code when participants discuss any conversations they may have had with their healthcare team about cost.

Research Question	Code Name	Description
Tell me about any treatment that you have received for your cancer thus far (Chemotherapy, surgery, non-surgical localized therapy, radioembolization.	**Treatment**	Use this code when participants discuss any thoughts or comments about the kind of treatment plan they are engaged in.
What, if anything, else about the treatment that you would like to know	**Treatment questions**	Use this code when participants discuss any questions they have about their ongoing treatment.
**Communication Issues**		
Since your cancer diagnosis, what instances, if any, have there been when you DID NOT feel that your medical providers were communicating well with you?<br><br>What could have been done differently?	**HCP communication**	Use this code when participants describe conversations between patients and providers, or descriptions of how a medical provider communicated something with the patient. This could be positive or negative or neutral.
	**Appointments**	Use this code when participants describe the process for scheduling appointments with their HCPs.
Is there a provider whom you identify as the "main provider" with whom you communicate for your liver cancer or whom you would contact if you needed help? Who is that provider?<br><br>Tell me about any experiences contacting this provider?	**Main provider id and communication**	Use this code when participants discuss thoughts or comments about who they see as their main health care provider and what it is like contacting and communicating with said provider
Tell me anything that you might want to know about what to expect in the future?<br><br>Prognosis, survival, cancer-related symptoms: Did your doctor talk to you about any such things?<br><br>Some people who are diagnosed with cancer want to be sure their "affairs are in order." Has this been something you have been thinking about?	**HCP communication future**	Use this code when participants discuss thoughts or comments about any conversations they have had with their health care team regarding prognosis, survival rate, getting their affairs in order or any other instance of thinking and discussing what the future may look like.
Some people choose to talk to family/friends about these issues. Have your talked to anyone about these things?	**Family/friend communication future**	Use this code when participants discuss thoughts or comments about any conversations they have had with their family or friends regarding prognosis, survival rate, getting their affairs in order or any other instance of thinking and discussing what the future may look like.
**Daily Functioning**		
Many patients experience a change in their day-to-day activities as a result of their cancer or their cancer treatment. Have you experience any such changes?	**Changes to daily life**	Use this code when participants discuss thoughts or comments any changes that have occurred or they anticipate occurring due to the cancer treatment they are on. This could include work duties, spending time with family/friends, caring

Research Question	Code Name	Description
		for others, exercising, activities in and around the community, etc.
Tell me about any people who have been sources of support to you and helped you since your diagnosis<br><br>What types of things are they helping you with?	**Support**	Use this code when participants describe who, if anyone, is providing support to them during their cancer treatment. This could include family, friends, church, social workers, therapist, etc.<br><br>Also use this code when participants describe a lack of support or someone who is not providing support.<br><br>Use this code when participants discuss thoughts or comments about any kind of support they have received during their cancer treatment. This could include emotional support, providing transportation, ADLs, housework, meals, etc.
**Recommendations**		
	**Recommendations**	Use this code when participants describe any suggestions for how to improve care.
