# Supplementary material for: Qualitative evaluation of barriers and facilitators to hepatocellular carcinoma care in North Carolina
Source: PLoS One. 2023 Jun 22;18(6):e0287338. doi: 10.1371/journal.pone.0287338 (PMC10287003; doi:10.1371/journal.pone.0287338)
Supplement: Attachment — Submitted filename: Response to Reviewers.docx [file pone.0287338.s003.docx]

**Please ensure that your manuscript meets PLOS ONE's style requirements, including those for file naming.**

Modifications made to adhere with style requirements.

**We note that you have indicated that data from this study are available upon request. PLOS only allows data to be available upon request if there are legal or ethical restrictions on sharing data publicly.**

There are ethical restrictions on sharing a de-identified data set, as the data contain potentially sensitive information, including patient names, provider names, cancer diagnosis, home residence city and state, and site of medical care, all of which could enable identification of a participant. The participant consent form indicates that we will share de-identified excerpts in research manuscripts but not the entire transcript. As such, we cannot share these data publicly. The contact for the research ethics committee at UNC is: [ethicsandintegrity@unc.edu](mailto:ethicsandintegrity@unc.edu).

**As you will notice, the reviewers expressed a preference that the methods should be expanded upon, in order to better guide the reader - I encourage you to take up this suggestion.**

Thank you for this suggestion. Please refer to the itemized changes below for specific areas in which we have expanded our Methods section.

**I also agree with reviewer 2 that further discussion on facilitators/enablers and the possible interventions they support may be particularly relevant in ensuring uptake of your work, and I would suggest to be clear in which interventions should be implemented by whom.**

Thank you for pointing out the need to expand our discussion of facilitators and possible interventions. The key facilitators identified were: provider knowledge of HCC; clear, honest, timely communication; prognosis conversations; strong social support; and financial support (see Results and Table 2). We have expanded our Discussion section to highlight these facilitators and propose interventions that leverage these factors (lines 312-319, 330, 337, 344-347, 379-381 in tracked version). Additionally, we have added additional specificity to the first paragraph of the Discussion section regarding how to leverage social support with specific interventions (lines 286, 291-292 in tracked version).

**Additional Editor comments:**

*** Please add a description of the consent procedure into the methods section of the manuscript, and clarify why only oral consent was requested and whether it was witnessed/documented in any way.**

Because potential subjects were located across the state of North Carolina and interviews could be performed in person or by telephone, in-person contact between the potential subject and study team was not required. As such, we obtained informed consent via telephone, using a script approved by our Institutional Review Board. Consent was documented on an IRB-approved form signed by the study coordinator. Additionally, at the time of the interview, patients were again read a script regarding the voluntary nature of participation and given the opportunity to decline participation before proceeding with the interview. This additional information has been added to the Methods, Participants and Recruitment section (lines 138-145 in tracked version).

*** Please move the ethics review/approval to a separate paragraph in the methods section.**
Completed as requested.

**Reviewer #1: Thank you for the opportunity to review this manuscript. It is an important topic and patients' perspectives of care experiences are crucial in developing appropriate improvements in care delivery at both system and clinician level.**
We thank the reviewer for their thoughtful review of our paper and for the opportunity to improve the manuscript by addressing their comments below.

**- Overall: Please review the manuscript for statements that require references. See, for example, Line 88:** additional citations added**, Lines 126-7:** additional citations added**, Lines 237-238:** citation moved to the end of the sentence, as it applies to the entire sentence**, and Lines 244-251:** additional citations added. Uncited comments are the thoughts of the authors interpreting our research findings in the context of existing literature. We appreciate you bringing these gaps to our attention.

**- Overall: Please review the manuscript for use of double quotation marks. If these are quotes from participants or from the literature on this topic, they should be identified as such. If these are not quotes from participants or from the literature, then please put them in singular inverted commas to identify them as your use of colloquial language.**

In all but one case, these are direct quotes from participants. We added language to better identify them as such. The term charity care was taken out of quotes as this is an accepted term and not colloquial. Thank you for suggesting this change.

**- Patients and Methods [Participants and Recruitment]: Your recruitment strategy requires more explanation to help readers understand the rationale: you state you recruited until thematic saturation was reached (Lines117-118) but if you continued to recruit, you may have come across new themes in the new data.**

Thematic or conceptual saturation means no new insights or information are being found by the researcher. As the researcher hears similar occurrences over and over again, they become increasingly confident they have reached saturation and that additional data collection would not yield new information. We have added additional language and a citation (lines 147-149 in tracked version) to try to improve clarity regarding this accepted qualitative research strategy.

**- Patients and Methods [Data Collection Procedures]: It would be valuable to be able to see the semi-structured interview guide as an appendix or supplementary file.**

We are happy to provide this. We have uploaded it as a supplementary file and mentioned it in the methods section (lines 157 in tracked version).

**- Patients and Methods [Analysis]: Please indicate the foundations of the thematic analysis approach you took. For example, your description of your method appears to bring together a quantitative (positivist - see Boyatzis 1998 and Joffe 2011) and qualitative (interpretative - see Braun & Clark 2006) approach - you might refer to it as Applied Thematic Analysis (see Guest et al. 2012 & 2014).**

We have addressed this issue by including the description of analytic approach as “directed content analysis” and a citation for this approach. This description is now at the start of the analysis sub-section within the methods section (lines 168-182 in tracked version). See paragraph below.

**- Patients and Methods [Analysis]: Please provide more detail about what was in the codebook. You state "They developed topical codes from the guides" (Line 136). What were the guides?**

We have reworded the analysis section with the below paragraph to provide more detail on the development and use of the codebook. We are also including the final version of the codebook as a supplementary file to be uploading with the paper and mentioned it in the methods section (S2 Appendix).

*Directed content analysis* was applied to code transcripts using a codebook developed from the interview guide and notes taken during data collection. The initial codebook was independently applied by three senior qualitative researchers (RT, JCA, MV) to several transcripts. Codes were compared across the three researchers, and discrepancies were identified and reconciled through discussion and consensus. This heuristic process continued until replicability of coding occurred across researchers, at which point the codes and coding decisions were finalized. The final version of the codebook (See Appendix XX) was then applied to the remaining transcripts. After coding all transcripts, researchers generated a report for each code to create narrative summaries, identify emergent themes and sub-themes, and select illustrative quotes. Findings were organized to identify patient-perceived facilitators and barriers to accessing and navigating HCC treatment and care.*

*Citation for directed content analysis was added: Hsieh HF, Shannon SE (2005). Three approaches to qualitative content analysis. Qual Health Res 15(9): 1277–1288.

**- Patients and Methods [Analysis]: Please add a statement about how you are reporting the results. What constitutes a 'theme' in this study? Qualitative analysis is not necessarily about who said what most often, and it is in keeping with qualitative methodology to report results from the analysis of singular transcripts, but you have described thematic saturation, so it would be helpful to readers if you explained your choices. This is particularly important given that in the Results section you refer to "most", "some" and "other" in relation to participants' perceptions and opinions.**

Thank you for the opportunity to clarify. We agree with the reviewer that it is in keeping with qualitative methodology to report any results obtained in an interview. This project utilized a qualitative methodology, the semi-structured interview, to explore what and how participants experienced access to HCC treatment and care, so all participant perspectives, even if just stated by one participant, were considered themes. Semi-quantification words like “most” and “some” provide readers with some indication of how often a concept or theme appeared across interviews but without sharing numbers that could be misinterpreted as generalizable. We have added a statement of clarification and citation in the manuscript (lines 177-182 in tracked version).

**- Results [Participant Suggestions for Improvement/ Improve Timeliness and Coordination of Appointments]: Please explain or delete the interpretive statement: "seemed to have greater satisfaction with their overall care." On what basis have you concluded "seemed to"?**

Review of the transcripts revealed a correlation between satisfaction with care and having a primary point of contact. In contrast, patients without a clear point of contact were less satisfied with their cancer care. We have modified this sentence to read “Patients who identified one of their providers as their primary point of contact expressed greater satisfaction with their overall care” to try to emphasize that this was stated by patients (line 276 in tracked version).

**- Discussion: This is a well developed discussion of the themes. It leans towards advocacy in tone, so please review this section to ensure that all statements are derived from the findings or are referenced.**

Thank you for your feedback. We are attempting to combine existing literature with new data from our study and discuss implications for practice. We have modified the language and added citations as requested. Please let us know if you think further modifications are required to emphasize interpretation of findings and minimize advocacy.

**Reviewer #2: General comments:
This is a well written article with a clearly described aim and methods. I have only minor comments and suggestions.**
Thank you for your time in reviewing our manuscript and for the favorable review. We have tried to address all comments in the revisions as outlined below.

**Specific comments:
1. Page 10, Line 112 – the authors mention they purposively sampled participants – what was the sampling frame (e.g., characteristics) that was used?**

We used a purposive non-probability sampling strategy to try to reflect the diversity and breadth of the sample population, in this case considering characteristics including age, race, marital status, location of home residence, and socioeconomic status to try and reflect the patients who come to UNC Health for HCC treatment and care. Additional detail has been added to lines 130-134 in the tracked version.

**2. Please describe the conceptual framework used to guide the study, and specifically the interview guide and analysis.**

This exploratory qualitative study was guided by social determinants of health and access to healthcare conceptual frameworks to help identify facilitators and barriers to patient access to HCC care. A clarifying sentence and additional citation has been added to lines 153-155 in the tracked version.

**3. I feel the that the results would benefit from a content analysis approach in the reporting – for example, it would be useful to know how many ‘codes’ arose for each of the subthemes e.g., 7 participants mentioned something about ‘improved timeliness and coordination of appointments’. Percentages are not necessary as they would not be useful for qualitative analysis, but the numbers of patients who reported would give good context.**

We thank the reviewer for this inquiry and for the opportunity to share the reasons that we did not report numbers of patients mentioning each theme or subtheme.

The research design and data collection methodology were chosen in part to allow for interactive, participant-driven interviews precisely because there was a dearth of information on this research topic in general. As such, the researchers were taking a listening stance to learn more and more about the topic as they went from one interview to the next. Therefore, when the researchers learned something about a given topic(s) from the first few participants, the substance of the interview may have changed to either accommodate these new learnings and/or target another aspect of the main topic. It is inherent in this work that participants are not asked the same questions in the same order. While the interview guide serves to prompt questions of all participants, participants are used to help verify information learned from previous interviews and are encouraged both to speak from their own experience and to speak for others. The main takeaway: Using in-depth interviews to learn from participants as the study progresses means each interview may overlap with the others but may also have a slightly different focus and different content, which has direct implications for how many times a given code may be used during analysis, or more importantly, how many participants said X or Y or Z.

While our study team could generate a list of the codes and the frequency in which they appeared across the 19 transcripts, these frequency numbers would only represent how salient the codes were in terms of number of times they were used, not the meaning or significance of the codes (for instance, the Cost code may have been used more often than the Treatment code but does not mean it was the most important/salient code). Some codes perhaps exhibited less salience/frequency than others because interview questions pertinent to that code were asked to some participants more than others or because interview participants had less to say about a given issue or because the code proved difficult to identify in the natural language responses of the interview participants. For that reason, we respectfully feel that the addition of these frequencies would detract from rather than enhance our manuscript and reader understanding of our research findings.

**4. Some aspects of the results need more insight and explanation (e.g., Page 10, Lines 179-181; Page 12, Lines 220-222) – one line to describe a subtheme seems a little thin – is there any further insight that can be provided?**

All participant perspectives, even if just stated by one participant, were considered themes. We provided excerpts of direct quotes in Table 2, as the quotes provide the most rich information from the participants. While these leaves some parts of the results section with a single sentence, we intentionally attempted to limit editorialization of these comments to not detract from the participant perspective. Interpretation and explanation of these findings are included in the Discussion.

**5. Page 14, Line 252 – the authors mention routine screening for HCC and that majority of patients were not undergoing routine screening. Can they elaborate on this more? Is routine screening for HCC available in the US (e.g., similarly to breast cancer or colorectal cancer screening)?**

Yes, American Association for the Study of Liver Diseases recommends that adult patients with cirrhosis, who are at the highest risk for developing HCC, should undergo surveillance using abdominal ultrasound, with or without laboratory testing for alpha fetoprotein, every 6 months. Screening for HCC improves early detection, enabling receipt of curative-intent treatment and improving overall survival in at-risk patients. However, uptake of screening has been suboptimal, and increased awareness of HCC screening among patients and clinicians is needed for earlier detection and improved outcomes. A clarifying sentence and additional citation has been added to lines 303-305 in the tracked version.

**6. Discussion – the authors summarise and critique the barriers to care well, but I would ask for some more insight into the facilitators and how these might be used as a basis for interventions (see next point).**

Thank you for pointing out the need to expand our discussion of facilitators and possible interventions. The key facilitators identified were: provider knowledge of HCC; clear, honest, timely communication; prognosis conversations; strong social support; and financial support (see Results and Table 2). As noted above in response to the Editor, we have expanded our Discussion section to highlight these facilitators and propose interventions that leverage these factors (lines 312-319, 330, 337, 344-347, 379-381 in tracked version). Additionally, we have added additional specificity to the first paragraph of the Discussion section regarding how to leverage social support with specific interventions (lines 286, 291-292 in tracked version).

**7. In the Abstract, the purpose of the study is to inform “tailor[ing] interventions to increase access to appropriate therapy” – could the authors please provide some more insight in the Discussion as to how their study findings will be used to tailor or develop such interventions?**

Thank you for highlighting the need to be more specific about how we will leverage these findings to develop interventions to help patients. We have added a few sentences about how the creation of a statewide HCC navigator role would help leverage many of the facilitators to HCC care and help reduce some of these barriers (lines 312-319 in tracked version). In the last paragraph of the Discussion, we also point out the importance of “investment in system-based supports such as caregiver engagement, patient care coordination, and financial navigation” which directly address many of these facilitators and barriers. While these additional supports may sometimes be perceived as supplementary, our findings underscore that these supports are truly essential. We also point out the importance of receiving HCC care in a multidisciplinary subspecialty care team and leveraging an HCC navigator to help facilitate that care.
